# Imaging Contrast of Diamond Nanoparticle Structures on Different Supporting Films

**DOI:** 10.3390/molecules31193368

**Published:** 2026-09-22

**Authors:** Yuying Yang, Feng Jiang, Yueyun Li, Jie Zhang, Tianhui Wu

**Affiliations:** School of Medical Laboratory, Qilu Medical University, Zibo 255000, China; yangyuying0533@163.com (Y.Y.);

**Keywords:** transmission electron microscopy, graphene oxide, support film, nanodiamond, imaging contrast

## Abstract

Electron microscopy is essential for nanoparticle research because it provides the critical structural data of particle size, morphology, crystallography, and elemental composition that are essential for tailoring and for predicting nanoparticles’ optical, electronic, catalytic, and mechanical properties. The weak scattering from nanoparticles results in low contrast in electron microscope images. Therefore, observing and interpreting such images requires special care to avoid erroneous conclusions. This study investigates the imaging contrast of diamond structures on different supporting films. When the nanodiamond is overlapped with a carbon film, particle edge details cannot be explained, and the overlapping interference lattices produce a pattern of interference fringes that is much coarser than the original pattern. Single graphene oxide (GO) sheets, as the ideal support film, are highly electron-transparent for the study of nanoparticles. As a low-background and high-transparency support to identify the dominant structural information of the nanodiamond, GO is superior to carbon support film. Quantitative contrast analysis reveals that GO film improves the contrast-to-noise ratio (CNR) by a factor of 3.0 for 8 nm particles compared with conventional amorphous carbon films. The {111} lattice-fringe detection rate increases from 35% on carbon film to 85% on GO for the same particle size. These results establish GO as a quantitatively superior support film for high-resolution electron microscopy (HRTEM) imaging of sub-10 nm nanoparticles.

## 1. Introduction

Since Max Knoll and Ernst Ruska demonstrated the first transmission electron microscope in 1931 [1], transmission electron microscopy (TEM) has become a useful and powerful technique in terms of material structure characterization [2,3]. TEM has been used in the study of solid carbon for approximately 80 years, revealing the microstructure of these materials, such as carbon black [4,5], graphitic carbon [6,7], diamond [8], nanotube [9], and graphene [10]. The development of aberration-corrected transmission electron microscopy, which increases resolution, now allows for the direct imaging of atomic structures, and this capability has had a significant impact on carbon materials science [11,12].

Diamond nanocrystals, as one of the major branches of carbon materials, have wide applications that range from materials to medical sciences [13,14]. Diamond nanocrystals consist of structurally inhomogeneous polymorphs [15,16,17,18] and many different defects including but not limited to mixed and screw dislocations, twins, and stacking faults terminated by Shockley or Frank partial dislocations [19,20]. TEM is indispensable for characterization of diamond nanocrystals. TEM has been extensively applied to obtain a detailed understanding of the defect structure of diamonds and diamond-like structures [21,22]. It is because of these structural defects that diamond nanocrystals are thought to possess exceptional physical properties, such as the unprecedented hardness and stability of the nanotwinned diamond [23].

In the process of diamond microscopic imaging, the final detailed imaging quality is affected not only by the quality of the electron microscope itself, but also by the supporting film of the specimen. In TEM, the diffuse background scattering of the support film usually obscures the weak diffraction from an individual nanoparticle [24]. GO [25], only one carbon thick, is highly electron-transparent due to its low atomic number and two-dimensional nature, which gives low background in both imaging and diffraction modes. At the same time, the preparation technology of GO is relatively mature, and the preparation method is much easier than that of ultrathin carbon films. In our group, we prepared a large amount of GO with clear layers and few defects using a modified Hummers method [26]. Worthwhile objects of study in themselves, and with previously explored applications in other scientific areas [27,28], GO has obvious applications in TEM. Based on its characteristic structure, GO is a nearly perfect support film for nanoparticles and macromolecules.

In this research, we have successfully synthesized high-quality GO and used it as a support film to image the diamond structure. By comparison with other support films, its ultrathin and almost electron-transparent nature allow single diamond particles to be imaged without introducing effects from other particles that would produce deceptive results, and thus the image becomes easier to explain. Furthermore, it is more helpful for diamond structural model construction and simulation [29]. While previous studies have qualitatively demonstrated the advantages of graphene-based supports for TEM, a systematic quantitative evaluation of contrast enhancement for sub-10 nm particles is still lacking. In this work, we not only synthesize high-quality GO as a support film but also perform a comprehensive quantitative analysis—including CNR, edge intensity profiles, lattice visibility, and FFT-based detection rates—to benchmark the imaging performance of GO against conventional carbon films for detonation nanodiamonds of varying sizes (6, 8, and 10 nm). This quantitative framework provides objective criteria for selecting support films based on particle size and structural targets.

## 2. Results and Discussion

### 2.1. The Structure of GO

In our experiments, the diamond crystals were powders 5~10 nm in size, produced by the method of explosion. GO of few layers was produced with a modified Hummers method using 93% sulfuric acid to intercalate graphite, and structural defects were formed during this process. As is evident in the low-magnification electron microscope image (Figure 1a), GO prepared by the well-controlled concentration of sulfuric acid has the morphology of thin film. Because the structure of GO contains both *sp*^2^ and *sp*^3^ hybridization, the bending of the layer should be directly related to the angle of the carbon–carbon bonding [30]. Figure 1b shows the selected-area electron diffraction (SAED) pattern from the Figure 1a region of the film. The clear diffraction spots, whose six-fold pattern is consistent with a hexagonal lattice, are observed to display a characteristic crystalline order that is similar to those obtained from GO [31,32]. The inner 010-type reflections are more intense than the outer 110-type reflections, the diffracted intensities of which are consistent with monolayer GO [33]. Among the observed GO, the number of layers varied, but they had been disrupted by the original AB stacking sequence. As shown in Figure 1c,d, apart from the monolayer GO, overlapping GO layers with a corresponding torsion angle of 14.0° measured by the SAED pattern were also observed.

The torsion angle of 14.0° observed in Figure 1d arises from the random overlapping of two GO sheets during sample preparation, rather than a specific coincidence site lattice (CSL) configuration. Such overlapping regions could, in principle, contribute additional Moiré fringes at certain defocus values. However, in our HRTEM imaging of nanodiamonds, the particles were deposited on predominantly single-layer GO regions with minimal overlap, as confirmed by the SAED pattern (Figure 1b) and by the clean, uniform background of the GO support in Figure 4. The Moiré contribution from overlapping GO layers to the nanodiamond images is therefore negligible in this study.

### 2.2. Optimum Scherzer Defocus

Under the condition of accelerating voltage of 200 kV (no aberration correction), the primary means to determine the resolution in HRTEM imaging is point resolution. When the so-called Scherzer defocus is used to balance the fixed positive coefficient of spherical aberration (Cs) from the image-forming objective lens in TEM, the highest point resolution is obtained, which is given by:

$${∆f}_{Sch}=-1.2({C_{s}\lambda )}^{1/2}$$

where Cs is the spherical aberration coefficient, and λ is the relativistic wavelength of the electrons. At this defocus, all the beams will have nearly constant phase out to the ‘first crossover’ of the zero axis. This crossover point provides a means to indicate the limit for the straightforward interpretation of images and is defined by the first crossover of the contrast transfer function with the x axis [34]. ${∆f}_{Sch}\approx -$66 nm (Scherzer defocus) was calculated for optimum Scherzer defocus conditions, resulting in positive phase contrast, meaning that atoms would appear dark against a bright background. Comparing Figure 2b with Figure 2a,c, we can observe that the condition of optimum focus of the microscope (Scherzer defocus) renders a clearer presentation of the structural details of diamonds of different diameters than the condition of over- or underfocus.

In TEM, the defocus value Δ*f* is defined as the distance between the objective lens focal plane and the specimen plane. By convention, negative defocus (Δ*f* < 0) corresponds to underfocus, where the objective lens is weakened, and positive defocus (Δ*f* > 0) corresponds to overfocus. The Scherzer defocus, ${∆f}_{Sch}$ ≈ −66 nm, represents the optimal underfocus condition that balances the spherical aberration of the objective lens, yielding the highest point resolution and positive phase contrast (atoms appear dark against a bright background).

### 2.3. Film-Free Nanodiamond Microscopy

In the process of obtaining the nanostructure of diamond, the conditions of TEM as well as the choice of supporting films are equally important to the straightforward interpretation of images. This is because the diffuse background scattering of the thick support film always obscures the weaker diffraction from the smaller nanocrystal that has fewer carbon atoms. In practice, actual observations should be made in areas where the specimen is exposed and suspended on the holes of a lacey carbon film [35]. However, the diameter of nanodiamond is too small, making it difficult to image single particles on the holes of a lacey carbon film. Without a supporting film on the holes, nanodiamond particles cluster together, obscuring the diamond structure. In order to illustrate this further, Figure 2 shows an experimental electron microscope image of diamonds of different diameters. Without a support membrane, images of the 6, 8, and 10 nm nanodiamonds were acquired at their optimal defocus, underfocus, and overfocus conditions. Diamond particles coexist with other diamond particles, so that the particle edge details cannot be explained, as shown in Figure 2a1–c1,a2–c2. When two particles are superimposed, two overlapping interference lattices produce a pattern of interference fringes—as shown in Figure 2b3, marked by a red arrow—that is much coarser than the original pattern and is very sensitive to differences in lattice spacing and relative orientations. Due to severe aggregation and overlapping of the particles, the image quality was insufficient for analyzing their crystal structure. Therefore, the selection of a supporting film that can be used for imaging is very helpful for the observation of diamond structure.

Two types of twins can be identified in nanodiamonds: (1) co-axis twins (also known as parallel twins or growth twins), where the twin plane is parallel to the electron beam direction, and (2) lamella twins, where the twin plane is inclined relative to the beam direction. In Figure 2b3, the observed twin is a lamella twin, as evidenced by the Moiré fringe pattern arising from the overlapping {111} lattices. In contrast, the twin boundaries marked in Figure 2 are co-axis twins, where the {111} twin plane is viewed edge-on along the <110> zone axis. The twin plane is {111}, and the twinning rotation angle is 70.52° around the <111> axis, characteristic of a Σ3 coincidence site lattice in FCC diamond.

### 2.4. Imaging Nanodiamonds on Continuous Carbon Support Film

Among the different supporting films, continuous carbon support film is very commonly used to examine the contrast behavior of nanocrystal images. Similarly, on the conventional carbon film, we imaged the 6, 8, and 10 nm particles at their optimal defocus, underfocus, and overfocus settings. As shown in Figure 3, when the nanodiamond was overlapped with continuous carbon support film, bright contrast fringes were observed at the periphery of the nanodiamonds in the TEM images. For nanodiamond particles with diameters of 6, 8, and 10 nm, no analyzable crystallographic information was obtained along the characteristic zone axes. It is practically impossible for the nanodiamond to produce usable features for unambiguous identification. The contrast difference decreases because the continuous carbon support film gives rise to much stronger scattering than the nanodiamond. However, as can be seen from Figure 3a1–a3, the contrast difference re-emerges as the diameter increases. For microscopic imaging of large particles, owing to the higher density of projected potential from the particle onto the supporting film, the traceable contrast from the particle could survive through a somewhat thicker film. In the microscopic imaging of the structure of small particles, continuous carbon support film on the order of 15 nm in thickness is too thick compared to the diameters of nanodiamond (<10 nm), so that the diffuse background scattering of the continuous carbon support film obscures the weaker elastic scattering from the nanodiamond.

### 2.5. Imaging Nanodiamonds on GO Film

To examine the contrast behavior for diamond images, we chose GO of a two-dimensional nature as a nearly perfect support film. Like graphene, GO is almost electron-transparent due to its single-layer atomic thickness, with a thickness of 1.4 Å and low atomic number. As a result, graphene and GO have obvious applications in TEM not only as objects of study in themselves [36], but also as nearly perfect support films for other nanoparticles and macromolecules. In addition, the preparation process of GO is much simpler than that of graphene. At the same time, GO is highly electron-transparent even in comparison to the thin-film carbon support. As expected, if film becomes even thinner, the image contrast due to the nanodiamond would become more apparent. This point is further affirmed by the HRTEM lattice image of nanodiamond on GO shown in Figure 4. To systematically investigate the size-dependent morphology and optimal imaging conditions, we acquired a series of through-focus TEM images for nanodiamonds with varying diameters (6, 8, and 10 nm) deposited on the GO membrane. Figure 4 shows contrast due to three nanodiamond particles of different sizes: particle (1), 6 nm in diameter; particle (2), 8 nm in diameter; and particle (3), 10 nm in diameter. The nanodiamond particles can be clearly resolved against the weak background of the GO sheet. According to the electron microscope images, a Σ3 (using the coincidence-site lattice notation) twin boundary of diamond structure viewed in a <110> direction is very common in nanodiamond [37]. The twin boundaries are indicated by red line segments in Figure 4. A clear dependence of both contrast and apparent size on the defocus value was observed for all particle sizes. Notably, nanodiamonds exhibited the most pronounced variation in visibility with focus, becoming clearly discernible only at specific defocus conditions. The contrast and sharpness of all particles were found to be highly sensitive to the defocus condition.

In particular, by comparing Figure 3 and Figure 4, we can observe that nanodiamonds deposited on the GO membrane exhibited superior contrast and clarity compared to those on conventional amorphous carbon films under identical imaging conditions. The nanodiamonds displayed markedly better-defined lattice fringes and a sharper interface against the GO substrate. This improvement is attributed to the atomically thin and crystalline nature of GO, which reduces phase noise and improves contrast, unlike the thicker, amorphous structure of conventional carbon films. Using GO as a low-background and high-transparency support, it is thus possible to identify the dominant structural information of the nanodiamond through conventional HRTEM. While the previous images illustrate the qualitative improvement, a rigorous quantitative assessment is necessary to establish the statistical significance and size-dependent nature of the contrast enhancement. We therefore conducted systematic measurements of four key imaging metrics: contrast-to-noise ratio (CNR), edge intensity drop, lattice fringe visibility, and FFT-based {111} detection rate.

### 2.6. Quantitative Comparison of Imaging Contrast on Different Supports

To move beyond qualitative observation, we systematically measured the CNR, edge intensity drop, lattice visibility, and {111} detection rate for nanodiamonds deposited on GO and amorphous carbon films.

#### 2.6.1. Contrast-to-Noise Ratio

Figure 5a summarizes the CNR values obtained from 20 particles per condition. For 8 nm particles, the CNR on GO (3.9 ± 0.6) was three times higher than that on carbon film (1.3 ± 0.3, *p* < 0.001). Notably, the CNR for 6 nm particles on GO (2.6 ± 0.5) already exceeded that for 10 nm particles on carbon film (1.8 ± 0.5), demonstrating that GO effectively shifts the detectable size limit toward smaller particles. A complete summary of CNR values for all conditions is provided in Table 1.

#### 2.6.2. Edge Sharpness 

The improved CNR on GO suggests better particle edge definition, which we further examined through line intensity profiles. Line profile analysis (Figure 5b) revealed that the intensity drop from particle center to edge was 69 ± 8% on GO, compared with only 35 ± 9% on carbon film for 8 nm particles, indicating that the particle–support interface is much sharper on GO. This improvement is attributed to the reduced background scattering from the atomically thin GO membrane. The edge intensity drop rates (EIDR) for all conditions are also listed in Table 1.

#### 2.6.3. Lattice Fringe Visibility and FFT Detection Rate

Beyond edge sharpness, the ability to resolve lattice fringes is critical for structural analysis (Figure 5c,d). We therefore quantified the visibility of {111} planes and their detectability in FFT patterns. The visibility of {111} lattice fringes (Figure 5c) increased monotonically with particle size on both supports, but the values on GO were consistently higher: for 8 nm particles, LV reached 0.42 ± 0.06 on GO versus 0.19 ± 0.03 on carbon film (*p* < 0.01). Consistent with this, the FFT detection rate of {111} reflections (Figure 5d) was 85% for 8 nm particles on GO, but only 35% on carbon film. For 6 nm particles, the detection rate dropped to 15% on carbon film, yet remained at 60% on GO, underscoring the advantage of GO for imaging ultrasmall nanocrystals. The correlation between LV and particle diameter suggests a linear relationship on both supports, with slopes of 0.0532 nm^−1^ (GO) and 0.0499 nm^−1^ (carbon), indicating that GO not only improves absolute visibility but also enhances the size-dependent contrast sensitivity.

Collectively, these quantitative data provide unambiguous evidence that GO support films significantly outperform conventional amorphous carbon films in terms of CNR (3.0× improvement for 8 nm particles), edge definition (2× sharper), and lattice information recovery (2.5× higher detection rate for 8 nm particles), particularly for particles below 10 nm in diameter. The statistical significance of these differences (all *p* < 0.01) confirms that the observed advantages are robust and not due to random variation. The quantitative superiority of GO is evident from the above metrics.

### 2.7. Stability of GO Under Electron Beam Irradiation and Its Practical Implications

A legitimate concern regarding the use of GO as a TEM support film is the susceptibility of its oxygen-containing functional groups to electron-beam-induced reduction, as noted in our experimental observations and previously reported in the literature [24]. At an accelerating voltage of 200 kV, inelastic scattering events can knock out oxygen atoms or desorb hydroxyl/epoxy groups, leading to partial deoxygenation of the GO membrane. We address this concern from three perspectives: temporal window, practical performance, and trade-off versus graphene, demonstrating that the partial instability of GO does not undermine its utility as a TEM support film under routine imaging conditions.

#### 2.7.1. Temporal Window for Imaging 

Under our low-dose imaging conditions (electron flux < 100 e^−^·Å^−2^·s^−1^, typical single-exposure time of 1–2 s), the accumulated electron dose per image is <200 e^−^·Å^−2^. At this dose level, previous in situ studies have shown that GO retains its structural integrity and macroscopic sheet morphology without noticeable shrinkage or hole formation during the first 10–20 s of continuous exposure [36]. This time window is more than sufficient for acquiring a single HRTEM image, including focusing and aberration tuning, which typically require less than 10 s of beam exposure on the region of interest. Therefore, for routine imaging applications where each particle is exposed only once, the beam-induced reduction of GO does not compromise the imaging quality or the validity of the contrast measurements reported here.

#### 2.7.2. Performance Under Practical Conditions 

Importantly, even if partial deoxygenation occurs, the resulting structure—often described as partially reduced GO (rGO)—retains its atomically thin, two-dimensional morphology. The thickness remains essentially unchanged (a single carbon layer), and the primary benefit for TEM imaging—namely, minimal background scattering due to the low projected mass density—is preserved. In fact, the contrast enhancement we observed (CNR = 3.9 ± 0.6 on GO vs. 1.3 ± 0.4 on carbon film for 8 nm particles) reflects the actual imaging conditions during our experiments, which already include any beam-induced changes. The quantitative superiority persists despite partial reduction, confirming that the support remains highly effective even in its partially modified state.

#### 2.7.3. GO Versus Graphene: A Practical Trade-Off 

Pristine graphene is chemically more stable and structurally more uniform than GO. However, the choice of support film in a typical materials or biomedical laboratory involves multiple practical considerations beyond stability alone. The preparation of high-quality, large-area graphene films requires chemical vapor deposition (CVD) followed by wet or dry transfer onto TEM grids—a process that is time-consuming, low-yield, and technically demanding. In contrast, GO can be prepared in any standard chemistry laboratory using a well-established modified Hummers method, yielding large quantities of single-layer sheets within a few days [26]. The aqueous dispersibility of GO enables simple drop-casting onto TEM grids without any transfer step, making it far more accessible for routine use. For researchers whose primary goal is rapid structural screening of nanoparticles rather than ultra-high-vacuum or in situ heating experiments, the ease and reproducibility of GO preparation outweigh the marginal stability advantage of graphene. We therefore sugggest GO as a practically optimal support film for routine nanoparticle HRTEM studies, rather than claiming it to be the electronically or structurally ideal one under all conditions.

In summary, the partial instability of GO under the electron beam does not undermine its utility as a TEM support film, provided that low-dose imaging conditions are adopted and images are acquired within the stable temporal window. The quantitative contrast advantages we have demonstrated are real and reproducible under actual operating conditions. For the vast majority of routine nanoparticle characterization tasks, GO offers an efficient combination of performance, simplicity, and accessibility.

## 3. Materials and Methods

GO was produced using a modified Hummers method with concentrated sulfuric acid. In brief, firstly, graphite powders (1.0 g) were placed in a flask under stirring in an ice bath. Then, under vigorous agitation, KMnO_4_ (5.0 g) was added slowly while keeping the temperature of the suspension lower than 20 °C. Successively, the flask was transferred to a water bath of temperature 20 °C ± 2 °C and stirred continuously for about 48 h. Next, 30 mL of water was then added into the solution, and it was stirred for an additional 5 min; 5 mL of H_2_O_2_ was then added, after which the color of the solution would turn from dark brown to light yellow. Finally, an additional 60 mL of water was added into the flask to obtain GO. Then, a 0.02 mg/mL GO suspension was prepared and ultrasonicated for 2 h to obtain a single-layer or few-layer GO suspension.

One drop of GO suspension is placed on an appropriate TEM grid and dried prior to the addition of the nanodiamond to be studied. GO support films can be suspended directly onto 400-mesh TEM grids without any aid. High-resolution electron microscopic images were acquired from electron-transparent areas of the residue protruding into the holes of the GO film and lacey carbon film. The GO support films used in this work were predominantly single-layer, as confirmed by SAED (Figure 1b) and by the low, uniform background in HRTEM images (Figure 4). The presence of oxygen-containing functional groups renders the GO surface hydrophilic, enabling uniform dispersion of nanodiamond particles from aqueous suspension. The GO was prepared by the same modified Hummers method as in our previous work [26], where the layer structure and defect density were characterized in detail.

HRTEM observations were performed with an FEI Tecnai G2 F20 S-TWIN electron microscope (FEI Company, Hillsboro, OR, USA) with a single-tilting stage at an accelerating voltage of 200 kV. The point resolution of the microscope, determined by the Scherzer defocus condition, is 0.24 nm, and the lattice resolution (information limit) given by the damping function is 0.14 nm. Images were obtained without introducing any objective aperture. TEM images were obtained using a Gatan SC-1000 Orius CCD camera (Gatan Inc., Pleasanton, CA, USA).

We proceeded then to image analysis and quantification. All HRTEM images presented in this work were acquired without any objective aperture. No filtering, denoising, or background subtraction was applied. Only global brightness and contrast adjustments were performed uniformly across the entire image using DigitalMicrograph (Gatan Inc., Pleasanton, CA, USA, version 3.4) to enhance visibility for publication. The red line segments and arrows in Figure 2 and Figure 4 are graphical annotations added for clarity and do not represent any image processing. Original, unmodified images are provided in Supplementary Figures S1 and S2. For each support film condition (continuous carbon and GO) and each particle diameter (6, 8, and 10 nm), 20 individual particles were analyzed. The contrast-to-noise ratio (CNR) was calculated as CNR = |Īp−Īf|/σf, where Īp is the mean intensity of the particle center (a circular ROI of 3–5 nm in diameter), Īf is the mean intensity of the adjacent support film, and σf is the standard deviation of the film intensity. Line intensity profiles were extracted along the particle diameter to evaluate edge sharpness. Lattice fringe visibility (LV) was measured from the intensity modulation of {111} planes as LV = (I_max_ − I_min_)/(I_max_ + I_min_), where Imax and Imin are the peak and valley intensities of adjacent fringes, respectively. Fast Fourier transform (FFT) patterns were computed from 5 nm × 5 nm regions containing the particle; the presence of {111} reflections was scored independently by two observers. All results are reported as mean ± standard deviation (SD). Statistical significance between support film conditions was assessed using a two-tailed Student’s *t*-test (Welch’s correction for unequal variances), with *p* < 0.05 considered significant. For the detection rate (DR), which is a binary outcome (detected/not detected), the results are expressed as a percentage with the standard error of the proportion: SE = √[*p*(1 − *p*)/*n*] × 100%, where *p* is the detection rate and *n* = 20 is the number of particles per condition. Statistical comparisons of DR between support films were performed using the two-tailed Fisher’s exact test, with *p* < 0.05 considered significant.

Single-layer GO possesses a hexagonal (trigonal) crystal structure with the space group P6/mmm (No. 191) and lattice constants a = b = 2.46 Å, c = 6.70 Å, α = β = 90°, γ = 120°. The in-plane C–C bond length is approximately 1.42 Å. Nanocrystalline diamond (cubic carbon) has a face-centered cubic (FCC) Bravais lattice with the space group Fd3m (No. 227) and lattice constant a = 3.567 Å. The {111} interplanar spacing is d_111_ = 2.06 Å, and the {111} twin boundary corresponds to a Σ3 coincidence site lattice with a rotation angle of 70.52° around the <111> axis.

## 4. Conclusions

In summary, we have systematically and quantitatively compared the HRTEM imaging performance of GO and amorphous carbon support films for detonation nanodiamonds ranging from 6 to 10 nm. Our quantitative measurements demonstrate that GO provides a statistically significant 3.0× improvement in contrast-to-noise ratio for 8 nm particles, approximately 2× sharper edge profiles, and a 2.4× higher detection rate of {111} lattice fringes for 8 nm particles. These advantages are particularly pronounced for particles below 10 nm, where conventional carbon films fail to provide interpretable structural information. Although GO undergoes partial reduction under the electron beam, its practical advantages in fabrication, hydrophilicity, and particle dispersion make it a superior choice for routine nanoparticle TEM studies. This quantitative framework establishes GO as a reliable platform for sub-10 nm crystalline particle analysis and provides objective criteria for support-film selection in HRTEM.

## Figures and Tables

**Figure 1:**
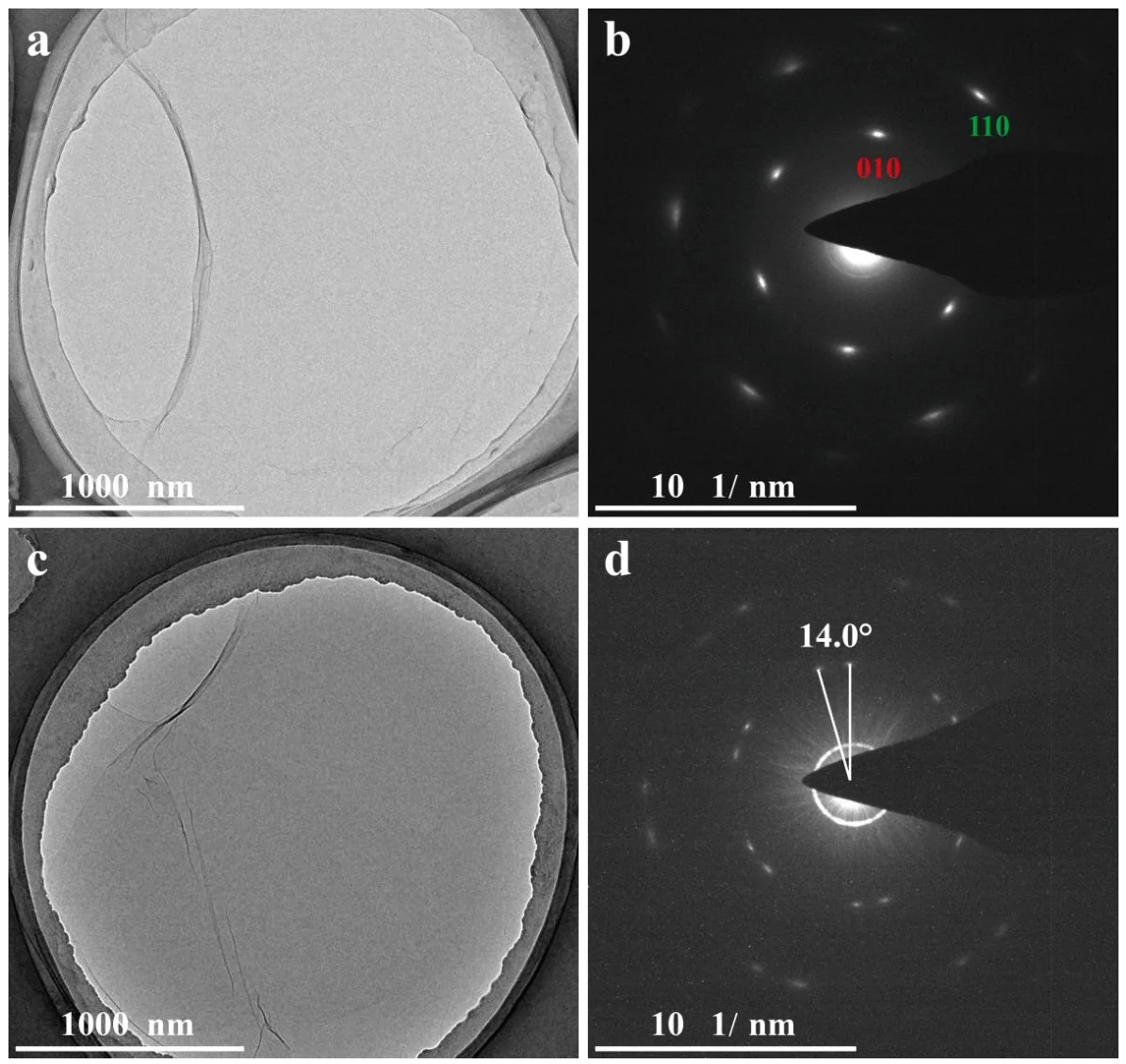
(**a**) Single-layer GO structure. (**b**) The corresponding SAED pattern of single layer GO. The inner 010-type reflections are more intense than the outer 110-type reflections. (**c**) The structure of bilayer GO. An overlapping GO sheet is visible, and the torsion angle is 14.0° measured by the SAED pattern (**d**).

**Figure 2:**
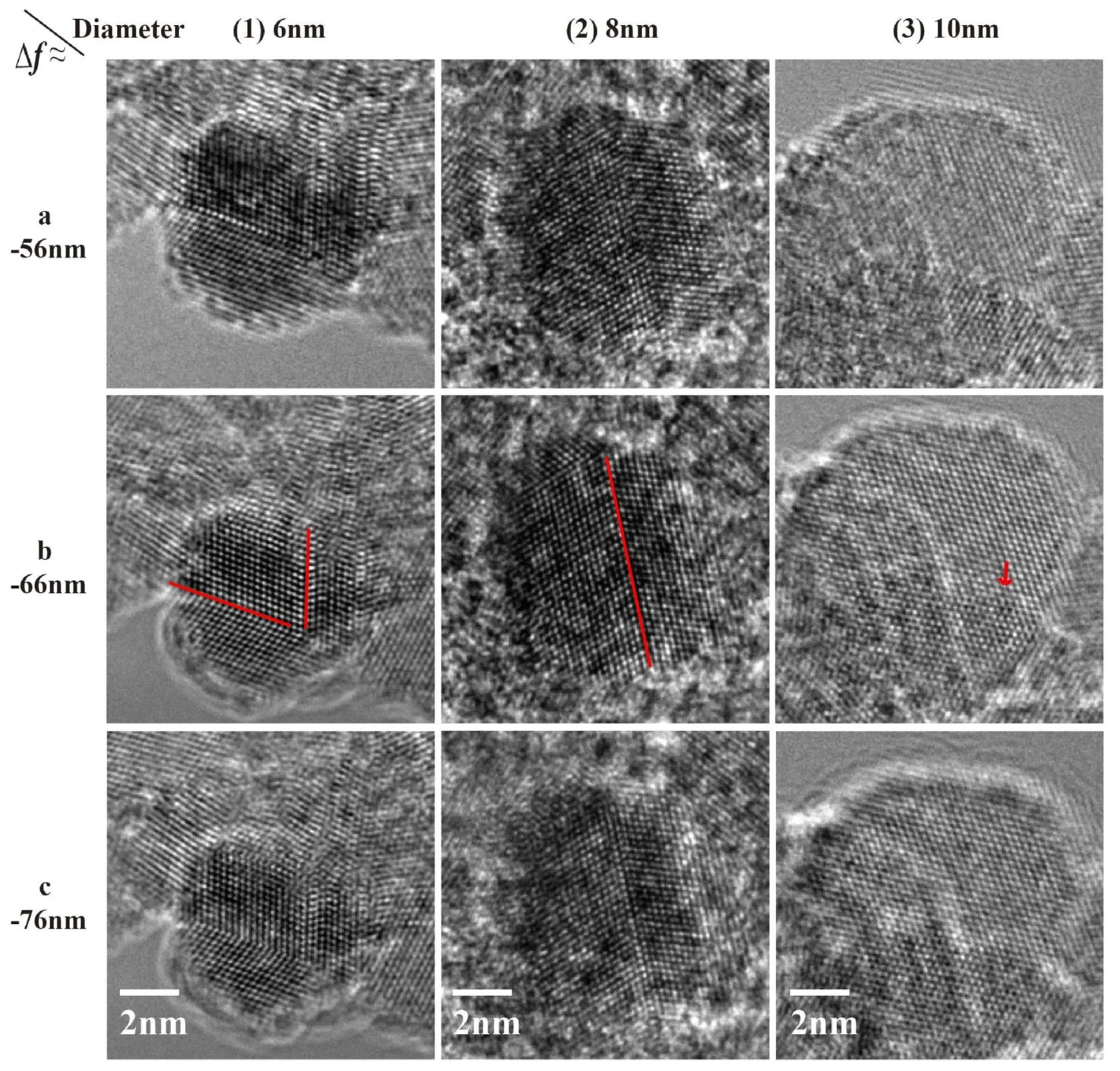
Micrographs of diamond of diameters 6 nm (1), 8 nm (2), and 10 nm (3) recorded under different conditions of focus. Under or over Scherzer defocus (**a**,**c**), the structural details are not clear. (**b**) Scherzer defocus is the condition of optimum focus of the microscope. (**b3**) Image of two overlapping interference lattices, marked by a red arrow, whose pattern is much coarser than the original pattern. {111} twin is very common in diamond. {111} twin boundaries are marked in red lines.

**Figure 3:**
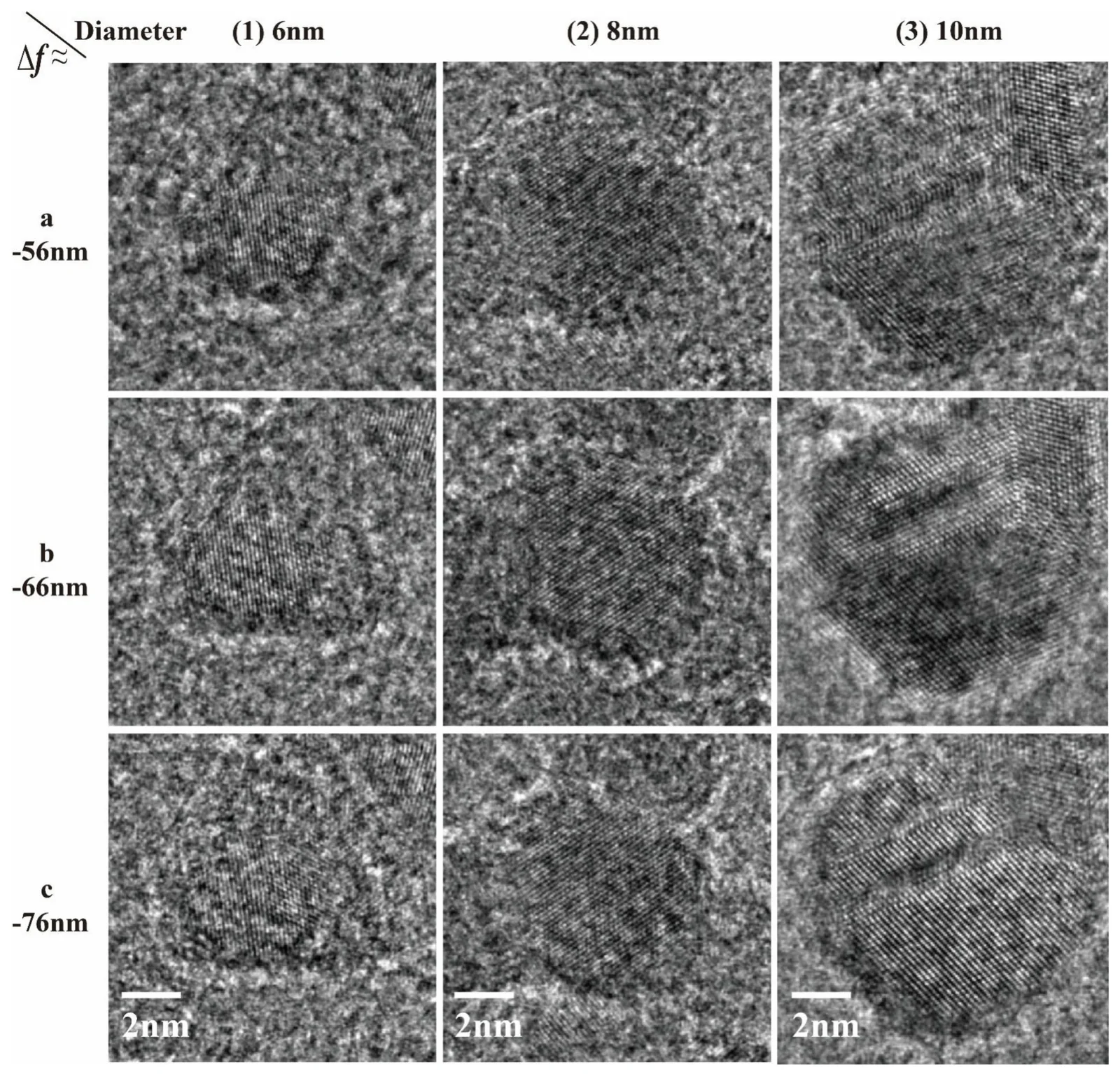
Micrographs of diamond of diameters 6 nm (1), 8 nm (2), and 10 nm (3) overlapped with continuous carbon support film. (**a**) Underfocus (Δ*f* ≈ −56 nm) and (**c**) overfocus (Δ*f* ≈ −76 nm); under these conditions, the structural details are not clear. (**b**) Scherzer defocus (Δ*f* ≈ −66 nm) is the condition of optimum focus of the microscope. The contrast difference is diminished because the continuous carbon support film gives rise to much stronger scattering than the nanodiamond. As seen in Figure 3b, the contrast difference re-emerges as the diameter increases.

**Figure 4:**
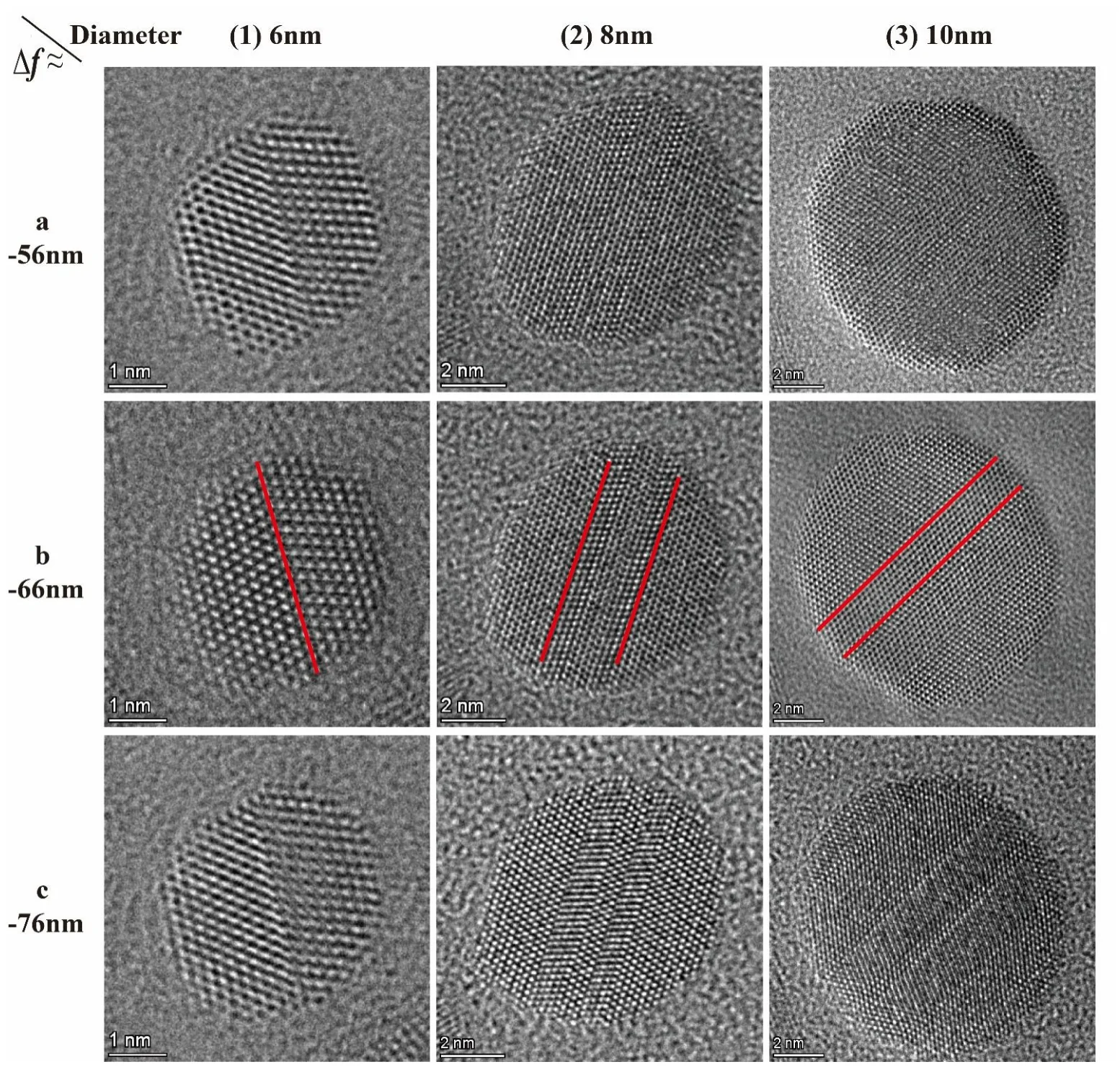
Micrographs of diamond of diameters 6 nm (1), 8 nm (2), and 10 nm (3) overlapped with GO film. (**a**) Underfocus (Δ*f* ≈ −56 nm) and (**c**) overfocus (Δ*f* ≈ −76 nm); under these conditions, the structural details are not clear. (**b**) Scherzer defocus (Δ*f* ≈ −66 nm) is the condition of optimum focus of the microscope. GO is highly electron-transparent. The nanodiamond particles can be clearly resolved against the weak background of the GO sheet. The red line segments in (**b**) indicate Σ3 {111} twin boundaries.

**Figure 5:**
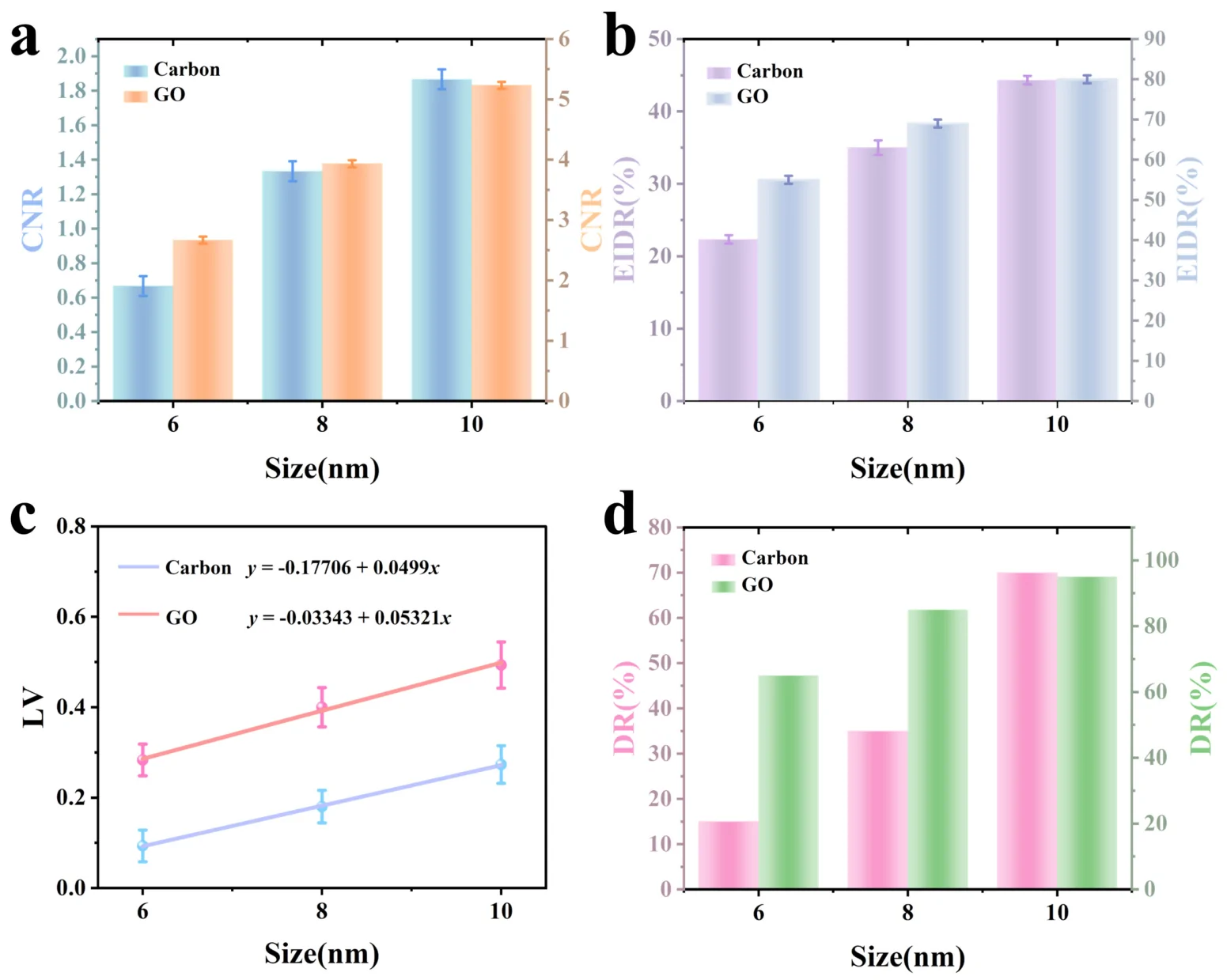
Quantitative comparison of imaging metrics for nanodiamonds on amorphous carbon and GO support films. (**a**) CNR of nanodiamonds with diameters of 6, 8, and 10 nm deposited on carbon (blue bars) and GO (orange bars) films. Data are presented as mean ± SD (*n* = 20 per condition). *p* < 0.001 (two-tailed *t*-test with Welch’s correction) between supports for each particle size. (**b**) EIDR of nanodiamonds on carbon and GO films. The EIDR values for GO are approximately twice those of carbon films across all particle sizes, indicating sharper particle–support interfaces on GO. (**c**) Correlation between LV of {111} planes and particle diameter on carbon (blue) and GO (orange) supports. Solid lines represent linear fits with 95% confidence bands. Slopes: 0.050 nm^−1^ (carbon, R^2^ = 0.996) and 0.053 nm^−1^ (GO, R^2^ = 0.999). (**d**) Detection rate of {111} reflections in FFT patterns for particles on carbon (pink) and GO (blue bars). Error bars represent the standard error of the proportion (*n* = 20 per condition). The detection rate increases from 35% on carbon to 85% on GO for 8 nm particles.

**Table 1:** Quantitative imaging metrics of nanodiamonds on different support films.

Support	Size (nm)	*n*	CNR	EIDR (%)	LV	DR (%)
Carbon	6	20	0.6 ± 0.3	22 ± 8	0.10 ± 0.03	15 ± 8
Carbon	8	20	1.3 ± 0.4	35 ± 9	0.19 ± 0.05	35 ± 11
Carbon	10	20	1.8 ± 0.5	44 ± 10	0.29 ± 0.03	70 ± 10
GO	6	20	2.6 ± 0.5	55 ± 9	0.30 ± 0.04	60 ± 11
GO	8	20	3.9 ± 0.6	69 ± 8	0.42 ± 0.06	85 ± 8
GO	10	20	5.2 ± 0.7	80 ± 7	0.42 ± 0.06	95 ± 5

Data are presented as mean ± standard deviation (SD) based on *n* = 20 particles per condition. CNR: contrast-to-noise ratio; EIDR: edge intensity drop rate; LV: lattice visibility of {111} planes. DR: detection rate of {111} reflections. For the DR, errors represent standard error of the proportion (SE = √[*p*(1 − *p*)/*n*] × 100%). Statistical comparisons between support types for each particle size were performed using a two-tailed Student’s *t*-test with Welch’s correction for CNR, EIDR, and LV, and Fisher’s exact test for DR. All comparisons showed *p* < 0.01, except for DR of 6 nm particles (*p* < 0.05).

## Data Availability

The original contributions presented in this study are included in the article/Supplementary Materials. Further inquiries can be directed to the corresponding author.

## Supplementary Materials

The following supporting information can be downloaded at: https://www.mdpi.com/article/10.3390/molecules31193368/s1, Figure S1. Original, unmodified HRTEM images of film-free nanodiamonds with diameters of 6 nm (1), 8 nm (2), and 10 nm (3) recorded at different defocus conditions: (a) underfocus (Δf ≈ −56 nm), (b) Scherzer defocus (Δf ≈ −66 nm), and (c) overfocus (Δf ≈ −76 nm). Corresponding to Figure 2 in the main text. No red lines or arrows were added to these original images; Figure S2. Original, unmodified HRTEM images of nanodiamonds with diameters of 6 nm (1), 8 nm (2), and 10 nm (3) deposited on GO support film, recorded at (a) underfocus (Δf ≈ −56 nm), (b) Scherzer defocus (Δf ≈ −66 nm), and (c) overfocus (Δf ≈ −76 nm). Corresponding to Figure 4 in the main text. No red lines were added to these original images.
