# Supplementary material for: Imaging Contrast of Diamond Nanoparticle Structures on Different Supporting Films
Source: Molecules. 2026 Sep 22;31(19):3368. doi: 10.3390/molecules31193368 (PMC13666105; doi:10.3390/molecules31193368)
Supplement: Supplementary file 1 [file molecules-31-03368-s001.zip › molecules-4468743-supplementary.pdf]

Imaging Contrast of Diamond Nanoparticle Structures on Different Supporting Films

Yuying Yang †, Feng Jiang †, Yueyun Li, Jie Zhang, and Tianhui Wu *

School of Medical Laboratory, Qilu Medical University, Zibo 255000, China; yangyuying0533@163.com (Y.Y.); jf15615736365@163.com (F.J.); liyueyun71@163.com (Y.L.); zhangjie@qlmu.edu.cn (J.Z.)

* Correspondence: wutianhui@qlmu.edu.cn

† These authors contributed equally to this work and should be considered co-first authors.

Image Processing Statement

All HRTEM images presented in this work were acquired without any objective aperture. No filtering, denoising, or background subtraction was applied. Only global brightness and contrast adjustments were performed uniformly across the entire image using DigitalMicrograph (Gatan) to enhance visibility for publication. The red line segments and arrows in Figures 2 and 4 of the main text are graphical annotations added for clarity and do not represent any image processing.

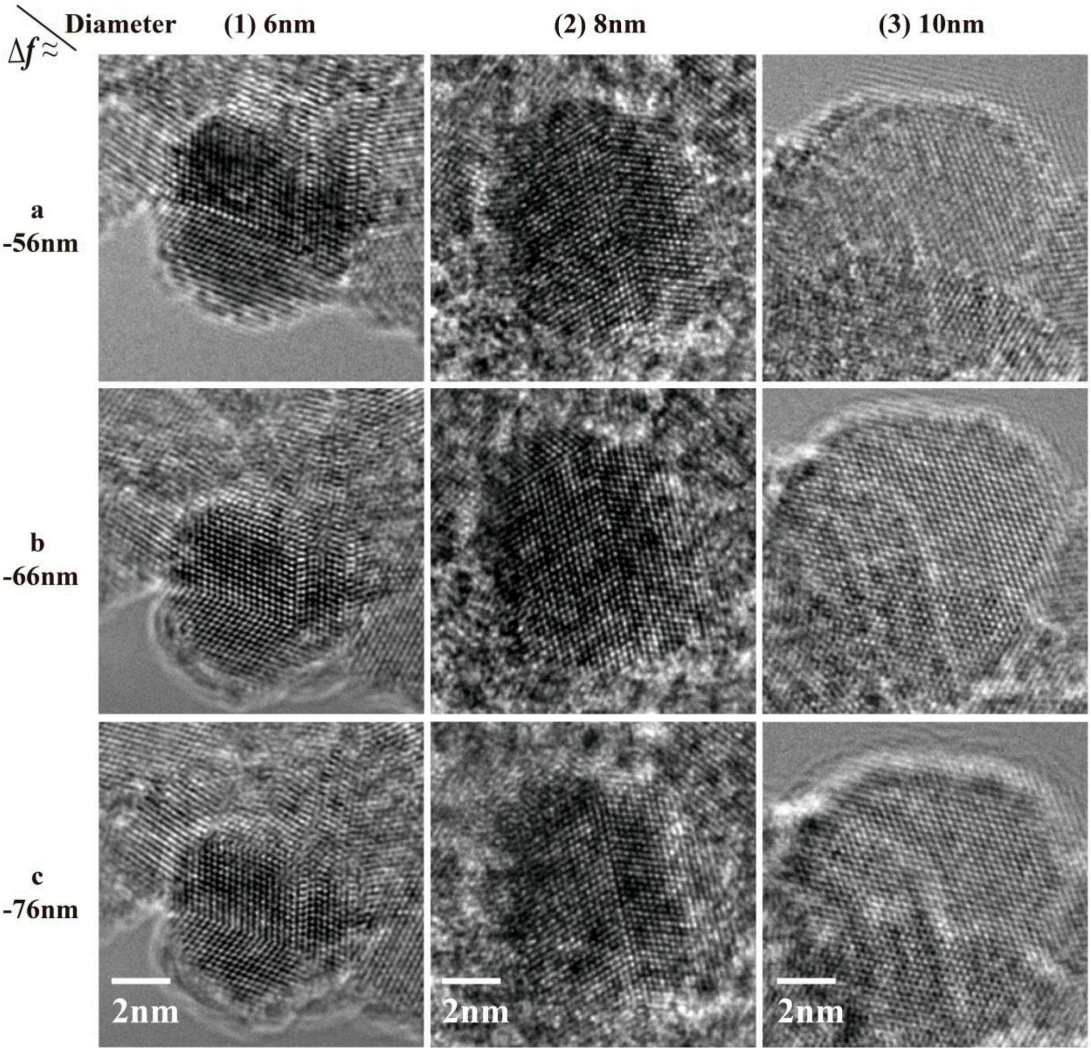

Figure S1. Original, unmodified HRTEM images of film-free nanodiamonds with diameters of 6 nm (1), 8 nm (2), and 10 nm (3) recorded at different defocus conditions: (a) underfocus ($\Delta f \approx -56$ nm), (b) Scherzer defocus ($\Delta f \approx -66$ nm), and (c) overfocus ($\Delta f \approx -76$ nm). Corresponding to Figure 2 in the main text. No red lines or

arrows were added to these original images.

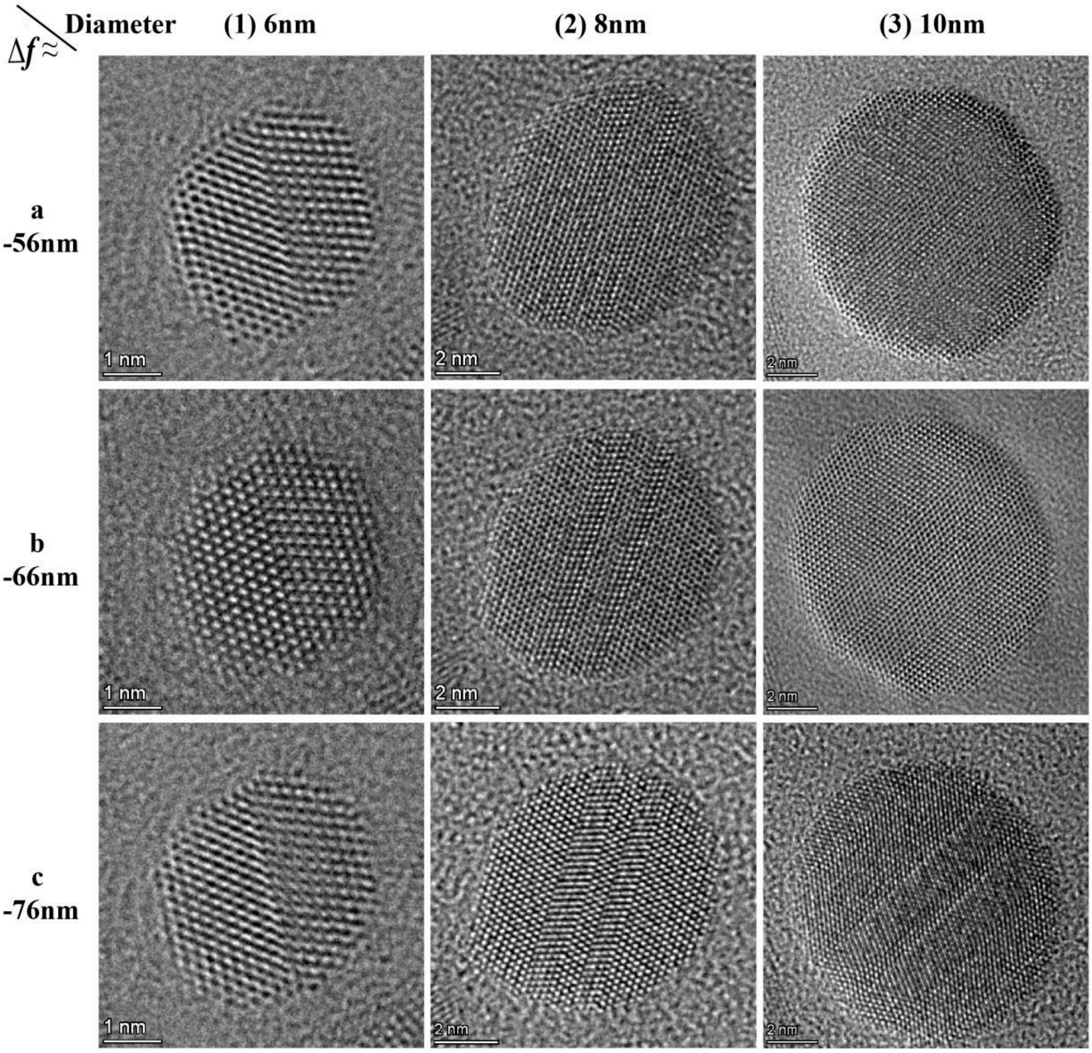

Figure S2. Original, unmodified HRTEM images of nanodiamonds with diameters of 6 nm (1), 8 nm (2), and 10 nm (3) deposited on GO support film, recorded at (a) underfocus (Δf≈−56 nm), (b) Scherzer defocus (Δf≈−66 nm), and (c) overfocus (Δf≈−76 nm). Corresponding to Figure 4 in the main text. No red lines were added to these original images.
